# Great ape infants’ face touching and its role in social engagement

**DOI:** 10.1007/s10071-025-01931-8

**Published:** 2025-02-05

**Authors:** Beatriz Felicio, Kim A. Bard

**Affiliations:** 1 Department of Experimental Psychology, University of São Paulo, Av. Prof. Mello Moraes 1721, C. Universitária, Sao Paulo, SP Brazil; 2 University of Michigan–Dearborn, Dearborn, USA

**Keywords:** Joint attention, Social cognition, Social touch, Chimpanzees, Humans, Infancy

## Abstract

Touch has a key role in the social development of infant primates and in the regulation of social interactions, even so, there’s a rarity of studies on infants’ use of social touch. In this work, we document chimpanzee infants and human infants’ touching of other’s faces, a behavior already described in wild capuchin monkey infants, and investigate possible functions of infants’ social touch. A strength of this study is that we sampled chimpanzee and human infants from three different social ecologies each. Each infant was observed naturalistically, in their everyday environments. In 36 h of observation, we found 269 touch events, specifically 222 face touches and 47 head touches. We found significant differences between groups, within species. Face touching occurred preferentially with adult females in all groups, and preferentially in prosocial contexts, although the most preferred contexts differed across groups. A unifying concept was that almost all infant face touching occurred during joint attention events. We interpret this as the ability of 1-year-olds to use face touching as a behavioral marker of mutual engagement during bouts of triadic connectedness, that is when they engage together with a social partner about an object or an event. In this study, we document an understudied behavior of young chimpanzees and humans, one that is not only part of prosocial interactions, but one that may function to highlight infants’ active role in engagement with another, while they together engage in triadic connectedness.

**Supplementary Information:**

The online version contains supplementary material available at 10.1007/s10071-025-01931-8.

Developmental psychologists have pointed to the role of mutual gaze in establishing human infants’ engagement in social interactions. “Mutual gaze can function as the occasion setter or the marker of the peak of the communication when vocal greetings and smiles occur (Bullowa 1979; Stern 1974; Trevarthen and Aitken 2001).” (Bard et al. 2005, p.616). This type of behavioral marker of mutual engagement is prevalent in the cultural contexts of Western, middle-class parents, with their emphasis on distal caregiving interactions. It has been proposed, however, that infants’ involvement in social interactions can be evidenced by more tactile communication, which is prevalent in the cultural contexts of rural communities, small scale communities, and/or those practicing more proximal caregiving interactions (e.g., in humans: Keller 2007; Takada 2020). Indeed, touch and other tactile forms of intersubjectivity can highlight mutual engagement, and may have a long evolutionary history (Bard 2009).

Joint attention, for Western, middle-class samples, involves infants’ coordinating their visual attention to objects with their visual attention to caregivers (e.g., Bakeman & Adamson 1984). Joint attention is an early form of social cognition that marks the developmental origin of shared intentionality, which is a combination of joint intentionality and joint attention with shared, cooperative goals (e.g., Tomasello 2019). Bard et al. (2021) broadened the definition of joint attention to apply cross-culturally and across species, labelling this decolonized form as triadic connectedness. This inclusive definition allows for when joint attention occurs via various non-visual modalities of infant-partner engagement, with various culturally meaningful topics, and with either infant or partner as initiator. For example, the broadened definition includes mutual engagement through touch or body contact, and includes shared topics, such as dancing. Bard et al. (2021) found that 1-year-old infants spent a majority of their time engaged with others about something (~ 65% of observed time), which was as true of chimpanzee 1-year-olds as it was of human infants. We agree with Bard et al. (2021) that triadic connectedness can function as a way for infants to learn about objects (e.g., in Western, middle-class settings), and, also, as a way for infants to learn about social activities (e.g., social roles and develop social skills). There is diversity in the relative proportion of triadic connectedness events with these two different functions within each species, and no evidence of species differences. Here we investigate the extent to which face touching may be involved in the mutual engagement that is an essential element of triadic connectedness, especially for those infants experiencing more proximal (tactile) than distal (visual) caregiving interactions.

Social touch can communicate emotion and information between two individuals (Hertenstein 2009) and it is involved in a variety of social interactions (Jablonski 2020). In the wild, infant capuchin monkeys (*Sapajus libidinosus*) softly touch other individuals on the face during affective interactions, especially in lip-smacking and grooming contexts (Felicio et al. 2024). This understudied behavior is an example of touch initiated by an infant as young as two months old. Although several studies support the importance of touch by others for the social development of infants (e.g., in chimpanzees: Bard 1994; in all primates: Bard 2009; and in humans viewed cross-culturally: Keller et al. 2009; Mantis et al. 2014; Meehan and Hawks 2013; Jean and Stack 2009; Takada 2020), there continues to be a paucity of investigations into the infant’s role in social touching.

It is known that chimpanzee infants, as well as adults, can interact with others using touch gestures (e.g., Bard et al. 2019). The touch gesture (which could be made with hands, feet, limbs, mouth, or lips, and directed to various body parts) occurs predominantly in affiliative contexts but seems not to contain any specific information. Although touch may be involved in some well-studied gestures (e.g., food begs, see below), gestures that are labelled as ‘touch’ contain a relative lack of distinction about properties, such as context, body part used, etc. (Bard et al. 2019). Both chimpanzee and orangutan infants, around 1 year of age, show food begging gestures in which the infant’s hand is directed to the mouth of the social partner in requesting the sharing of food, although touching of the lips or chin may, or may not occur (Bard 1992; van Lawick-Goodall 1968). With our new focus on proximal interactions (e.g., changing definitions of joint attention to triadic connectedness to allow for tactile engagements: Bard et al. 2021), we investigate infants’ role in social touching, especially touching of the face (independent of its use as a gesture, e.g., in referential communication). To date, there are few studies that have focused on infant face touching of others and its behavioral context in great apes. Most of the literature on social touch is focused on humans, and by adults, in particular. There is a research gap concerning the active role of infants and on the specific function of infants touching on the face of others.

## Current Study

In this article, we use the infant face touching behavior described in the study of Felicio et al. (2024) with wild capuchin infants, to document the occurrence, context, and partner selection in the touching by human and chimpanzee infants on the faces of others. Felicio et al. (2024) investigated face touching by free-living infant capuchin monkeys (*Sapajus libidinosus*) over the first three years of their lives. Using the hand to actively touch the face of another is part of the species’ social repertoire. Infants use it as a response to the engagement initiated by their partner in specific affiliative contexts, such as lipsmacking and grooming, and is accompanied by eye contact. In capuchins, this behavior has its peak during the second month of life when infants start to consistently engage with the physical and social environment (Araujo et al. 2024), showing its importance for early social interactions.

Here we investigate the face touching of chimpanzee (*Pan troglodytes*) and human (*Homo sapiens*) 1-year-olds. We observed their interactions in everyday settings, without any experimental manipulations. We did not know how often the behavior would occur, but given infants capuchins use their hands to touch the face of others, and chimpanzee infants use their hands in food-begging, we did expect to find instances of this behavior in both humans and chimpanzees. We explored the context in which face touching occurred to indicate possible functions of face touching in the clade of Primates. If face touching was not merely accidental contact, we expected it would occur more often during affiliative contexts. One-year-old chimpanzees and humans are becoming independently mobile, so they could direct this behavior to other social partners, in addition to the mother. It was possible that the particular area touched could be important, and so we described this as well.

We sampled human infants from three diverse settings, prototypical Western, middle-class settings near Universities, subsistence farming communities, and small-scale foraging communities. In this way, we can suggest the extent to which the role played by infant touches may be similar or differ across diverse human socio-ecologies. Similarly, and for the same reason, we sampled chimpanzee infants from three diverse settings, a Zoo in the UK, an enriched laboratory setting in Japan, and Gombe Nature Reserve, one of the long-term field sites in Africa.

First, we investigated whether infants touching the face was distinct from the more general head touching. We expected that touching would occur significantly and preferentially on the face compared to the head, and more often in prosocial contexts, if social touch plays a prosocial role. The preferential touching of the face by infants could indicate that they are already aware of the special status of the face in prosocial interactions, e.g., as the source of facial expressions. Although the face may be a more intimate and informative region than the head, there are also more physical features in the face (even though we included the ears as prominent physical features of the head) which could also be the source of more face touching than head touching. Alternatively, touches could be directed in general to all parts of the body, and there could be no difference in the rate of face touching and head touching.

Secondly, it is plausible that infants could use face touching as a special marker of communicative engagement. Studies in a variety of non-western, non-middle-class families have shown that body stimulation and touch can mark mutual engagements (e.g., Keller 2007; Takada 2020). Therefore, it is possible that infants’ touching of the face of a social partner could be used within the specific context of triadic connectedness, perhaps as an alternative to visual fixation, to emphasize or mark that the two partners are engaged together in their coordinated attention to an object or an event. The videotapes we used to score face touching had previously been scored for instances in which infants were engaged in joint attention and with the more inclusive term of triadic connectedness (Bard et al. 2021). If the function of primate infant face touching was related to triadic connectedness, we would expect to find significantly more face touching during bouts of triadic connectedness than when triadic connectedness did not occur. However, if infant face touching was simply part of general prosocial activity, then there would be no systematic relation with triadic connectedness.

The findings of this research can add a new dimension to our understanding of how young chimpanzees and human infants actively initiate and sustain mutual engagement in affiliative contexts. Considering the gap in the literature on social touch initiated by infants, this study will contribute to a deeper understanding of touches and the role they might play in primate social cognition.

## Methods

### Samples

This study used the same diverse samples as Bard et al. (2021). We believe that comparative studies should include various samples within species, e.g., between captive and wild groups, when using different species. Therefore, we sampled human and chimpanzee infants from three populations to understand if there were intra-specific variations in their developmental context. We observed the behavior of infants belonging to three different sociocultural contexts, given that interpersonal touch varies depending on status, culture, social class, context, location, etc. (e.g., Gallace and Spence 2010). It was important to not rely on the usual ‘two groups, two species’ type of comparisons, in which multiple variables are confounded with species, e.g., socio-ecological variables such as socialization practices, group composition, and parenting goals (e.g., Keller and Bard 2017; Leavens et al. 2019). With our diverse samples, we could determine the extent to which the range of outcomes for one species overlapped with the range of outcomes from the other species (see Bard et al. 2021 for more detailed information on this type of species comparison).

We selected videos of infants approximately one year old, previously collected by different institutions (Jane Goodall Institute Research Center, University of Portsmouth, and Primate Research Institute), most were used in Bard et al. (2021), and made available for this study with permission. There were groups for which we had exact infant ages, UK humans (Mean = 12mo, 8 days, Range = 11 mo, 18 days to 13 mo, 2 days), Gombe chimpanzees (Mean = 13mo, 3 days, Range = 12–15 months), PRI/Zoo chimpanzees (Mean = 12 mo, 3 days, Range– 11mo 12 days to 12 mo, 15 days). Ages for the Aka and the Nso human groups was approximately 1 year based on determinations by fieldworkers with extensive. specialized knowledge.

We coded infant touching in three groups of chimpanzees and three groups of humans. Details regarding the number of partners per infant, the date the videos were recorded, and the style of parental care for each population can be found in Table 1.

**Fig. 1:**
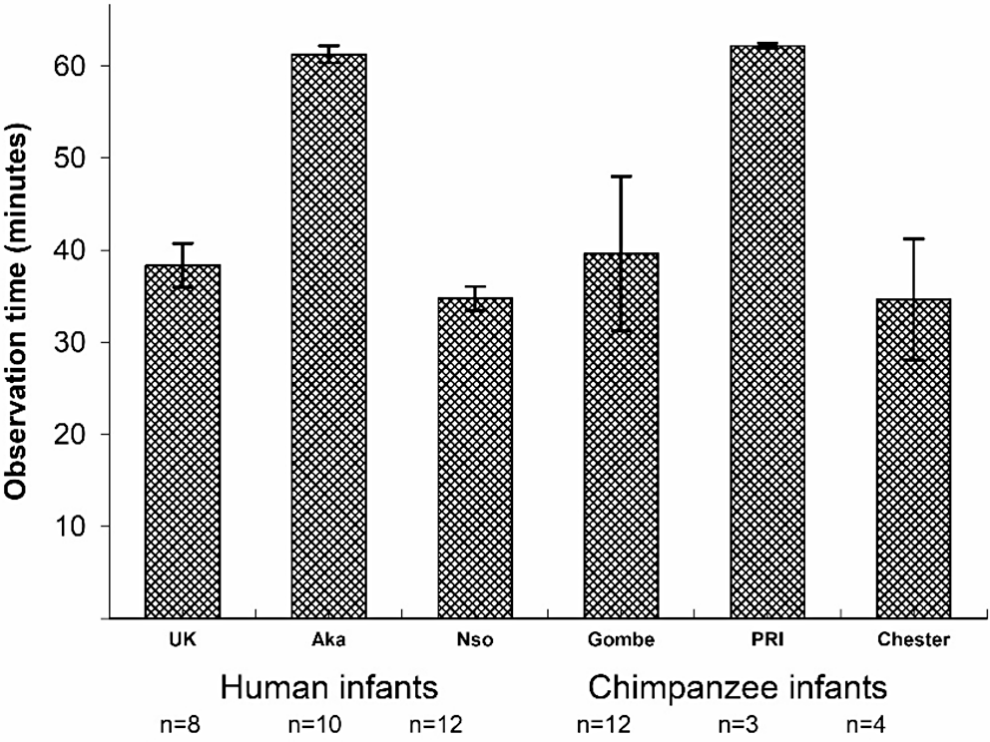
Observation time for each group, across species (Mean, SE shown in the bar charts, n indicates the number of individuals in each group)

There was a total of 12 h 13 min of videotaped observations for the 19 chimpanzee infants and 23 h 55 min of videotaped observations for the 32 human infants. There was no significant difference between the observation time of chimpanzee infants (Mean = 42.11 min; SD 25.11) and human infants (Mean = 44.53 min; SD 12.20); (Fig. 1: *F*(1, 49) = 0.265; *p* =.61, *partial eta squared* = 0.005). We did not want to truncate the time of the longer sessions and chose, instead, to compute rates of face and head touching.

**Table 1:** Demographic information of the dataset. (adapted from Bard et al. 2021)

Group (number of infants)	Video Dates	Caregiving Style	Physical Ecology; Social Ecology: (additional references)
*Human infants*			
UK (8)	2007–2008	Distal	Urban; nuclear family; SE England
Aka (10)	2010	Proximal	Mobile camps in Tropical Forest in Central African Republic; group of 25–35 individuals (mixed age, sex, & kinship: Hewlett and Roulette 2016; Meehan and Hawks 2013)
Nso (14)	2004–2005	Proximal	Highland Savannah in Cameroon; patriarchal extended family compounds (6–45: Keller 2007)
*Chimpanzee infants*			
Chester Zoo (4)	2006	Proximal	Indoor & outdoor enclosures in the UK; stable group of 27 chimpanzees of mixed age/sex/kinship (Ross et al. 2014)
PRI (3)	2001	Proximal	Indoor & outdoor enriched enclosures in Japan; stable group of 11 chimpanzees. Both mothers and infants had daily contact with a human (Matsuzawa et al. 2006)
Gombe (12)	1993–2003	Proximal	Tropical Rain Forest; fission /fusion structure of 3–61 individuals with mixed age/sex/kinship (Goodall 1986)

Of the total of 36 h of footage, we coded 269 touch events, specifically, infants used their hand 222 times to touch a social partner on the face (defined as the area between the ears and from the chin to the end of the forehead) and used their hand 47 times to touch a social partner on the head (including the ears, sides, and top of the skull). Detail of the location of touches is available in Table 1 of the Supplemental Material 2, but due to some small frequencies, we report touches only with reference to face and head locations. We coded touches on the head to serve as a comparison for touches to the face. If touches to the face were non-communicative, we would expect touches to the face to be approximately equal to touches to the head. We expected that touches on the face would be greater than touches to the head if the touches were communicative, as the partner would feel touches to the face more than touches to the head (in part due to differences in hair, and in part because of greater sensitivity of the face than the head). We did not make any predictions about species differences because we expected there to be meaningful intra-specific diversity in social touching in both species (see rationale in data analysis section below). Given the current evidence for triadic connectedness in human and chimpanzee infants (it occurred ~ 63% of the time, the same rate across species: Bard et al. 2021), we did not expect that there would be species differences in the relation of face touching with triadic connectedness.

For each touch of the infant’s hands, we identified the area of contact with the other individual’s face or head, the age/sex classification of the individual being touched, and the context in which the event occurred. All of the initial coding was conducted by BF. KAB reviewed all events, in which she verbally presented her coding after watching the videotaped event without knowledge of BF’s coding. Usually, these matched exactly with BF’s coding. When they differed, the event was reviewed by both coders until all details of coding were agreed upon. With this procedure, no reliability estimates were required, since the final agreement was 100% for all events. This information allowed us to define the broad social context of infant touches on the face and compare the phenomenon between groups and species.

For each touch, we coded the age/sex classification of the recipient. During coding, the possibilities were adult female, adult male, adolescent, juvenile, child, and infant. Although we had information on partner age in the chimpanzee groups, we did not have information on the age of partners in the human groups. Therefore, most of these judgements were based on size relative to the infant. We note that all adolescents that were touched were female (based on clothes for the humans and genitalia for the chimpanzees). Some of the age/sex classifications were infrequently observed and, therefore, for analyses, we collapsed them as shown in Table 2. Table 4 of the Supplemental Material 2 shows the original categories and reduced age/sex categories. In some analyses, we further collapsed these classifications, into the Adult female category versus all other partner categories.

**Table 2:** Interaction partners touched grouped for statistical analysis

Partner Code	Partners included
1	Mother, adult females, adolescent females
2	Father, adult males
3	Older siblings, juveniles, child
4	Infants

We coded infant touches within 19 possible prosocial contexts, two negative social contexts, and six contexts that were not social (these are listed and defined in Table 3). The contexts considered were of a wide variety of prosocial contexts, as we were not sure which would be most prevalent in our data. The contexts labelled as negative–social consisted of instances in which the infant was being restrained (e.g., prevented from traveling too far from caregiver, or to potentially dangerous locations), or there was low-level aggression (e.g., hitting or biting). Non-social contexts included instances where individuals were touched by the infant accidentally when the infant was engaged in non-social activity (e.g. moving an arm while stretching and accidentally touching the head of someone nearby).

**Table 3:** Contexts of facial or head touch (with observed frequency) with definition

Category	Context	Definition
Prosocial-Play	Play (123)	The infant touches another in the context of interactions with no evident purpose in a positive context (e.g. being tickled, lightly bitten, restrained, chased, sharing positive displays, or with objects).
Prosocial-Food share	Food beg/food share (24)	The infant touches another in the context of food, either the infant trying to obtain food in the possession of the partner or the partner trying to take food in the possession of the infant.
Prosocial-Groom	Groom/being groomed (18)	The infant touches another in the context of inspecting the skin or hair of the partner with their hands, or the context of the partner inspecting the skin or hair of the infant.
Prosocial-Rare	Comfort seeking/ being comforted (3)	The infant touches another while in distress, in an apparent attempt to gain whole body physical contact.
Prosocial-Rare	Hug (4)	The infant touches another while in torso-to-torso contact with the other.
Prosocial-Rare	Kiss /soliciting kiss (2)	The infant touches another while their or the partner’s lips are in contact with the face or head of the other, or the infant approaches the face of the other, resulting in a kiss afterward
Prosocial-Rare	Nursing (1)	The infant touches another while gaining access to the breast and sucks the nipple.
Prosocial-Rare	Greeting (1)	The infant makes a positive display directed to an individual when entering their shared space. The display can vary according to culture or species.
Prosocial-Rare	Appeasement (1)	The infant touches another when one or the other tries to soothe the emotions of the other after an aggressive or stressful interaction.
Prosocial-Rare	Submissive focal (2)	The infant offers the back of the hand or wrist in the direction of the face of the other (chimpanzees).
Prosocial-Other	Other social context (35)	The infant touches another in any other social interaction that seemed positive but does not fit the previous definitions.
Negative-social	Aggression (11)	The infant touches another in the context of hitting or biting.
Negative-social	Restraint (1)	The infant touches another in the context in which their mobility is restricted by another individual holding their body or limbs.
Non-social	Movement (9)	The infant touches another while moves her/his body either for locomotion or to adjust position.
Non-social	Arm movement (13)	The infant touches another while moves their arms only, without a particular aim or for a non-social purpose.
Non-social	Body support (15)	The infant touches another while using the body of another for assistance to stand or stay in place.
Non-social	Food (3)	The infant touches another when manipulating or consuming food on their own while others are close by.
Non-social	Solitary play (1)	The infant touches another while playing without partners, either with objects or by unusual locomotion (e.g., running around a tree, rolling on the ground).
Non-social	Startle to non-social stimuli (1)	Infant touches another when they get surprised by sudden movement, objects, the environment, or individuals of a different species.

For some analyses, we condensed the pro-social contexts, in part because some contexts occurred infrequently and in part because we had particular questions. For example, one question was whether face touching occurs differentially in the most frequently occurring contexts and we collapsed the seven rarely occurring pro-social contexts (comfort seeking/being comforted, hug, kiss/soliciting kiss, nursing, greeting, appeasement, and submissive focal) into Prosocial-Rare.

We transformed raw frequencies into rates for each partner touched, each touch context, and total face and head touches for each infant, which was possible because we recorded the exact duration of each event. The following formula was used for all rates: touch duration (in seconds) / total observed time of each individual, and multiplied by 600 (to obtain rate per 10 min). We chose to use 10 min to have more whole numbers in figures and statistics. For example, a rate of 1 per 10 min was preferred to the equivalent rate of 0.1 per minute.

To investigate one possible function of face touches in both species, we merged the face touching data with previously collected data on triadic connectedness (from Bard et al. 2021). This was possible because the time of each face touch was recorded, as well as the time of each triadic connectedness event. Bard et al. (2021) coded the presence (or absence) of triadic connectedness for each 10s interval in the video samples. Cross-classifying intervals of 10 s duration was the chosen methodology because (1) the focus was on specifying the components of triadic connectedness; (2) there was no need for frame-by-frame coding; (3) the coding was easy with a decision (of presence or absence) made every 10 s; (4) previous studies suggested that bouts of joint attention lasted less than 10 s; and (5) reliability was easier to assess (i.e., with Cohen’s Kappa) than with raw frequencies (Bard et al. 2021). For each touch event, we ascertained whether it occurred with an interval of triadic connectedness or not. Some touches either lasted longer than 10 s or otherwise occurred across intervals, and these were only counted in the first interval in which they occurred. Rarely more than one touch occurred within a 10 s interval and each of these was counted, as these touches were separate individual touches. A few touches occurred during portions of the video that had not been previously coded in the Bard et al. (2021) dataset. In these cases, KAB (the primary coder for Bard et al. 2021 dataset) conducted new triadic connectedness coding for at least a minute (6 intervals) surrounding the time of the face touch, without knowing in advance which interval(s) contained the face touch.

### Ethical note

This study is based on videotaped naturalistic observations. All the videotapes were recorded initially for other studies (for details see Bard et al. 2021 and acknowledgments) with appropriate institutional approvals (Jane Goodall Institute, University of Portsmouth, Washington State University, Vancouver, Osnabruck University, and Primate Research Institute) before the videotaping. All observations were naturalistic, everyday activities, conducted without constraints imposed by the investigators.

## Data analysis

Initially we used frequency data to assess the extent to which touch location (face versus head) was related to either touch partner or touch context (using a non-parametric chi-square: Siegel 1956), a likelihood ratio chi-square test (which has more power than a regular chi-square test when there are some small expected frequencies: Özdemir and Eyduran 2005). Then, using rates, we compared face versus head touching location for each of the six groups using repeated measures ANOVA. If there was a significant group effect or significant group by location interaction, we planned to conduct simple contrasts to identify which groups differed.

When we found a significant effect of group in any ANOVA, we conducted simple contrasts to determine which groups differed. We wanted to know if there were differences among the chimpanzee group and differences among the human groups (the two types of within species variation) and whether there was overlap between species. In the simple contrasts, we selected the Chester Zoo group as the chimpanzee referent and the UK group as the human referent because these groups represent commonly used samples in comparative (and development) psychology (Bard & Keller, 2024; Leavens et al. 2019; Vonk 2021).

Next we investigated whether the rate of face touches differed across groups (and species) as a function of the partner touched or as a function of the context. We ran two MANOVAs, one with the four options for partner (as reported in Table 2) and another one with the seven options for context (the contexts reported in the first column of Table 3). We report if there were significant group differences for each partner and each context. If there was a significant group difference, we ran a univariate ANOVA, with simple contrasts to determine which groups differed. We ran further ANOVAs to understand any significant interactions.

A major question was whether adult females were the preferred partner, in general, and so ran a repeated measures ANOVA to compare face touching rates directed to the adult female class versus all other partners (see Table 2). A second major question was whether the rate of face touching was higher in prosocial contexts, in general, and therefore we also ran a repeated measures ANOVA to compare face touching rates in prosocial (collapsing all prosocial contexts listed in Table 3) versus non-prosocial contexts (i.e., negative -social and non-social).

In these analyses, we compared species only if we did not find any within species differences and there was no overlap in any group across species. In other words, if we found a significant group effect and simple contrast revealed that there was a significant difference across the three groups of human infants or there was a significant difference across the three groups of chimpanzee infants, and/or there was an overlap across some groups of the two species, we refrained from conducting analyses comparing the species. Our rationale was that there could only be a consistent effect of species when the three sampled groups of a species did not differ from each other or all three groups of one species differed from all groups of the other species (there was no overlap across species). If not, then it would be inappropriate to consider that the three groups of humans (or chimpanzees) were all the same. For example, Bard et al. (2021) analyzed the predominant social partner for triadic connectedness. Adult females were the predominant partner for the UK human sample and for the PRI/Zoo and human-reared chimpanzee samples. For the Nso and the Aka human samples and for the Gombe chimpanzee sample, however, there was no predominant partner (the percent of events with adult female partners was not statistically different from the percent of events with other partners). Although an adult female was the most typical partner for one sample of human infants, it was not universal for all human infants, and therefore cannot be said to characterize the species.

It has been a problem in comparative (and developmental) psychology that outcomes from one group of humans (e.g., from WEIRD settings: Henrich et al. 2010; Nielson et al. 2017) have been considered the human norm, even when there is evidence of significant within species differences sufficient to dispel this assumption (see Chap. 1 in Bard et al. 2021 for review). Similarly, outcomes from single groups of chimpanzees, typically BIZARRE (Barren, Institutionalized, Zoo And other Rare, Rearing Environments: Leavens et al. 2019), have been erroneously considered the chimpanzee norm. It is our assumption that developmental experiences impact forms of communication and social cognition (it is social context that impacts social cognition), and when these experiences differ then social cognition and communication outcomes will differ, as well (see Bard & Leavens 2014; Leavens et al. 2019; for further discussion; and for evidence in within species differences, in human and chimpanzee infants, see e.g., Bard et al. 2021; Russell et al. 2011). It is only in those characteristics for which no significant within species differences were found, without overlap across the species, that we investigated between species differences.

Our final analysis investigated the extent to which infant touches to the face of partners was related to triadic connectedness events. The number of face touches that occurred within an interval of triadic connectedness was compared with the number that occurred when triadic connectedness was absent. If the behavior in an interval was not sufficiently visible to code triadic connectedness, then face touching was not counted (note that this occurred rarely - only 7 times - and only for the Gombe chimpanzees). The resulting face touch (yes or no) by triadic connectedness (yes or no) matrix for each group, with significance determined by a chi-square test, can be found in Table 6 of the Supplemental Material 2. We summarize the results in the text.

## Results

### Touch location

We investigated the extent to which there was an overall relation between the location of the touch and context with frequency data. There were too many cells with expected frequencies of less than 5 to consider all the contexts separately (Table 2 of the Supplemental Material 2). Therefore, we collapsed the contexts of Prosocial-rare and Groom/Being groomed into the Prosocial-other context, while retaining the contexts of Play, Food beg/food share, Negative-Social, and Non-social (Table 3 of the Supplemental Material 2). The likelihood-ratio chi-square (4) was significant, *p* =.024. This means that the contexts in which infants directed touches to the head differed significantly from the contexts in which infants directed touches to the face. The context in which the effect was strongest was Food Beg/Food Share, in which infants only touched the partner’s face, never the head.

**Fig. 2:**
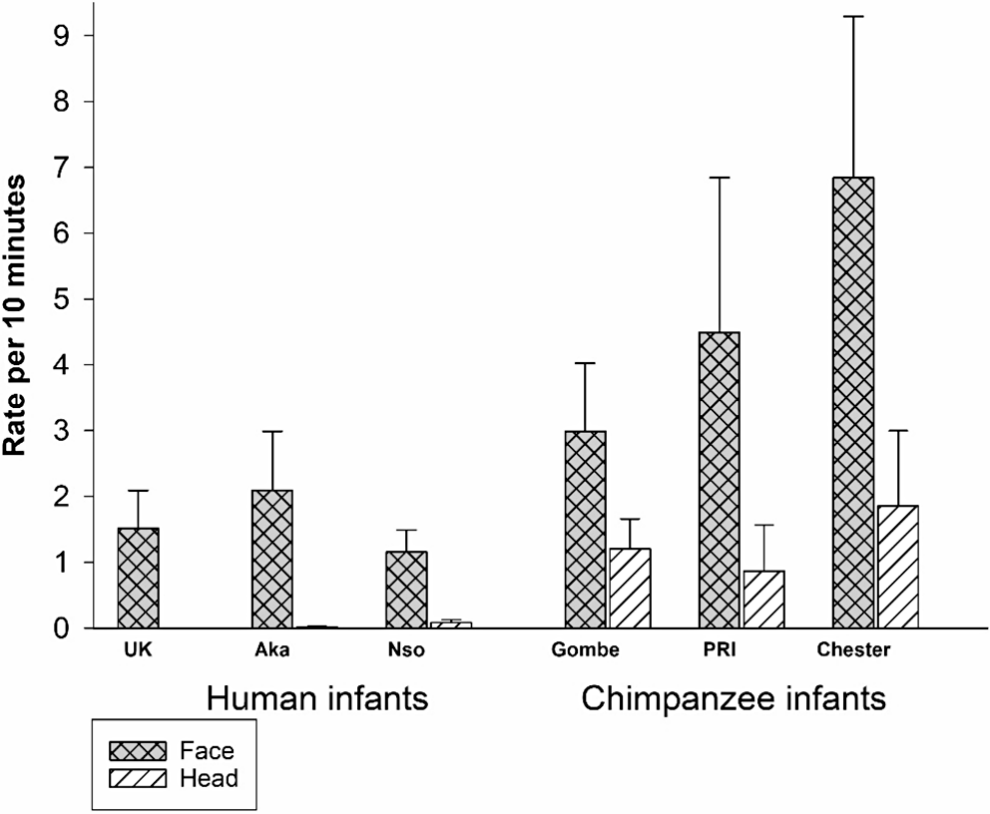
Infant face touching vs. infant head touching (rate per 10 min) for each of the 3 groups of chimpanzees and the 3 groups of humans (Mean *±* SE)

We found that the rate of face touching was significantly higher than the rate of head touching (Fig. 2), *F*(1,45) = 33.60; *p* <.001; *eta*_*p*_^*2*^ = 0.428. There was an overall main effect of group, *F*(5,45) = 4.369; *p* =.003; *eta*_*p*_ = 0.322 but no interaction between the touch location and group, *F*(5, 45) = 1.65; *p* =.166; *eta*_*p*_ = 0.155. We compared the groups with simple contrasts with Chester as the referent and found that infant touching rates did not differ between the Chester and PRI chimpanzee groups (*p* =.193), but the rates at Chester were significantly higher than all the other groups, i.e., versus Gombe (*p* =.023), versus UK (*p* <.001), versus Aka (*p* =.002) and versus Nso (*p* <.001). We conducted simple contrasts with the UK as a referent and found the UK humans differed significantly only from the Chester Zoo chimpanzees (*p* <.001) and did not differ significantly from the PRI chimpanzees (*p* =.09), the Gombe chimpanzees (*p* =.09), or the other human groups, Aka (*p* =.74) and Nso (*p* =.82). Because there was a significant difference within the chimpanzee groups (even though the human groups did not differ from each other), and overlap between some of the cross species groups, we did not conduct an ANOVA between species. In other words, face touching occurred significantly more often than head touching, and infant touching was significantly more frequent in the Chester chimpanzees than the other human and chimpanzee groups, which did not differ.

### Choice of partner

We considered whether there was a partner preference associated with touch location, using frequency data. Using the four categories of partners listed in Table 2, the likelihood-ratio chi-square (3) was significant, *p* <.001. Adult males and juveniles were touched on the face more often than expected, whereas infants were touched more often on the head than expected (Table 5 of the Supplemental Material 2).

Our next question was whether infants touched the face of adult females preferentially. We first ran a MANOVA with the 4 types of social partners (Table 2), which revealed (1) a non-significant trend for the omnibus effect of group, *F*(20,180) = 1.546, *p* =.071, *eta*_*p*_^*2*^ = 0.147; (2) a significant group difference in face touching of adult female partners, *F*(5,45) = 3.51, *p* <.01, *eta*_*p*_^*2*^ = 0.28, but (3) there were some groups without any instances of some of partner types (e.g., no face touching of adult males in the PRI and Gombe chimpanzee groups, and in the Nso human group; and 4) some groups in which particular age/sex partners were not available (i.e., there were no juveniles in either of the Chester and PRI chimpanzee groups, and there were no infants in the UK human group). Therefore, we combined rates for all non-adult female partners and re-ran the repeated measures ANOVA.

**Fig. 3:**
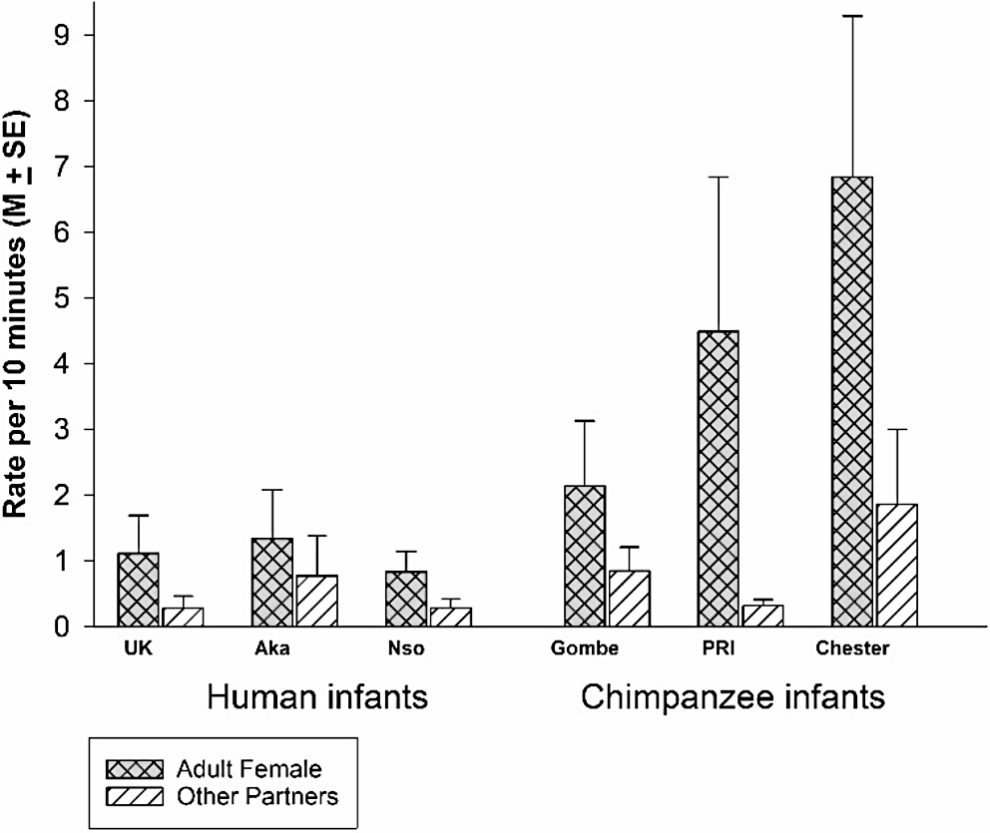
The rate of touching the face of an adult female compared to touching the face of all other partners for each of the 3 groups of humans and the 3 groups of chimpanzees

Infants touched the faces of adult females at significantly higher rates than touching the faces of all other partners, *F*(1,45) = 20.97, *p* <.001, *eta*_*p*_^*2*^ = 0.318 (Fig. 3). The groups differed significantly in overall rates of face touching, *F*(5,45) = 3.24, *p* =.014, *eta*_*p*_^*2*^ = 0.265, and there was a significant interaction between group and partner, *F*(5,45) = 2.829, *p* =.026, *eta*_*p*_^*2*^ = 0.239.

To understand the group differences in overall rates of face touching, we conducted simple contrasts (with Chester as the referent and then with UK as the referent). The Gombe chimpanzees and all the human groups had significantly lower rates than the Chester chimpanzees (vs. Gombe, *p* =.022; vs. Aka, *p* =.007; vs. Nso, *p* <.001; vs. UK, *p* =.003). The rate in the PRI chimpanzees did not differ from that of the Chester chimpanzees (*p* =.28). In comparison to the UK human infants, only the Chester chimpanzees had a significantly higher rate of face touching (UK vs. PRI, *p* =.11; vs. Gombe, *p* =.22; vs. Aka, *p* =.594; vs. Nso, *p* =.827).

To understand the group by partner interaction, we ran repeated measures ANOVA for each group separately. The difference between partners was not significant in any of the human groups, the UK infants, *F*(1,7) = 1.631, *p* =.242, *eta*_*p*_^*2*^ = 0.189, the Aka infants, *F*(1,9) = 0.307, *p* =.593, *eta*_*p*_^*2*^ = 0.033, or the Nso infants, *F*(1,13) = 2.412, *p* =.144, *eta*_*p*_^*2*^ = 0.156. The difference between partners was not significant for any of the chimpanzee infants, the Gombe infants, *F*(1,11) = 1.462, *p* =.252, *eta*_*p*_^*2*^ = 0.25, or the PRI infants, *F*(1,2) = 2.378, *p* =.23, *eta*_*p*_^*2*^ = 0.543, although there was a nonsignificant trend (with a large effect size) in the Chester chimpanzee infants, *F*(1,3) = 0.075, *p* =.057, *eta*_*p*_^*2*^ = 0.75. These analyses did not illuminate the interaction of partner by group.

To understand the partner by group interaction, we ran two univariate ANOVAs, one on rates of face touching in adult female partners and one on rates of face touching in non-adult female partners. There were significant group differences of rates of face touching to adult female partners. Simple contrasts in comparison with the Chester chimpanzee group revealed no significant difference from PRI (*p* =.281), but Chester had significantly higher rates than all the other groups (vs. Gombe, *p* =.008; vs. Aka, *p* =.002; vs. Nso, *p* <.001; vs. UK, *p* =.002). Simple contrasts in comparison with the UK group revealed a significant difference only from Chester, *p* =.002. Face touching rates to Adult Females in the UK infants were statistically indistinguishable from those in PRI, *p* =.09; in Gombe, *p* =.09, in Aka, *p* =.86, and in Nso, *p* =.81. There was not a significant main effect of group in rates of face touching to non-adult female partners, F(5, 45) = 0.510, *p* = 767, *eta*_*p*_^*2*^ = 0.054. Because there was an overlap between the species, and significant within species differences in the chimpanzees in the rates of face touching to Adult Females, we did not conduct an analysis by species.

### Contexts of face touching

**Fig. 4:**
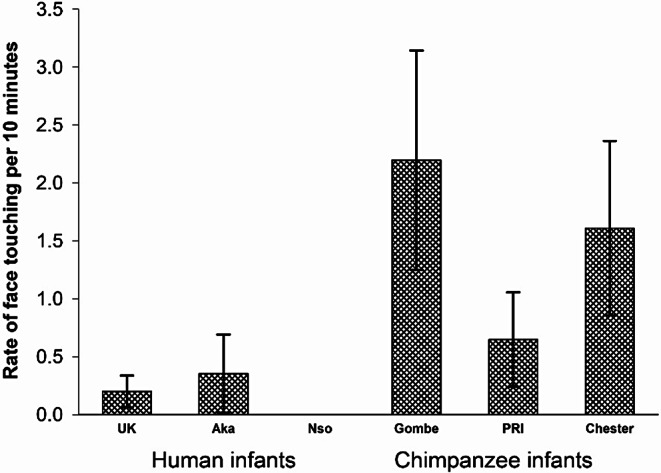
Rates of face touching during the context of Play for the 3 groups of humans and the 3 groups of chimpanzees

**Fig. 5:**
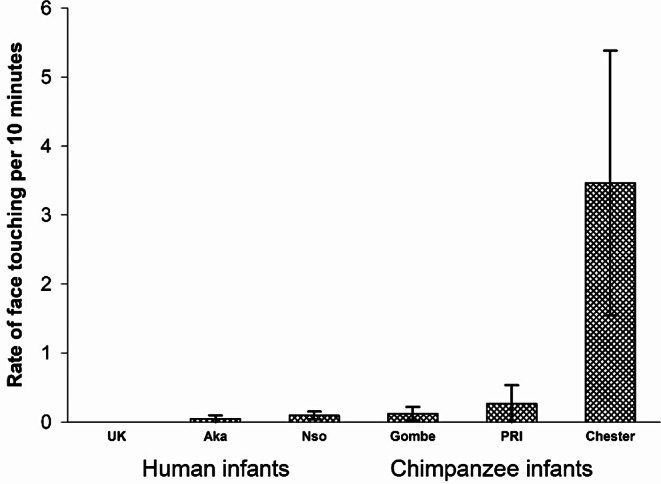
Rates of face touching during the context of Food beg/Food sharing for the 3 groups of humans and the 3 groups of chimpanzees

**Fig. 6:**
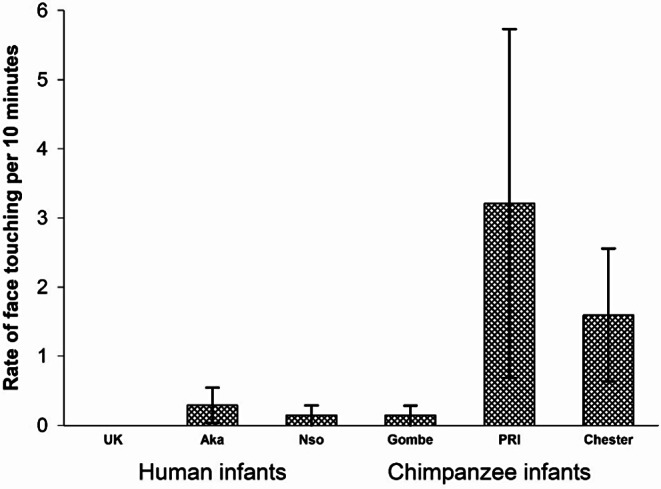
Rates of face touching during the context of Grooming/Being groomed for the 3 groups of humans and the 3 groups of chimpanzees

The next question was whether face touching occurred more in some contexts than others. We ran a MANOVA comparing the rates of face touching in the 7 contexts listed in Table 2 of the Supplemental Material 2 and found an overall significant effect of group, *F*(7,35) = 2.303, *p* <.001, *eta*_*p*_^*2*^ = 0.273. Specifically, there were significant group differences in the rates of infant face touching in the contexts of Play, *F*(5,45) = 2.614, *p* =.037, *eta*_*p*_^*2*^ = 0.225 (Fig. 4), Food beg/food sharing, *F*(5,45) = 8.450, *p* <.001, *eta*_*p*_^*2*^ = 0.475 (Fig. 5), and Grooming/Being groomed, *F*(5,45) = 4.736, *p* =.001, *eta*_*p*_^*2*^ = 0.345 (Fig. 6). There were no significant differences among groups in rates of face touching in the remaining contexts of Prosocial-Other, *F*(5,45) = 0.93, *p* =.47, *eta*_*p*_^*2*^ = 0.094, Prosocial-Rare, *F*(5,45) = 1.628, *p* =.172, *eta*_*p*_^*2*^ = 0.153, Negative-social, *F*(5,45) = 0.677, *p* =.64, *eta*_*p*_^*2*^ = 0.07, and Non-social, *F*(5,45) = 0.739, *p* =.598, *eta*_*p*_^*2*^ = 0.076.

In the context of play (with UK as a referent), simple contrasts revealed that the rate of face touching was significantly higher in the Gombe chimpanzees, *p* =.016, but all other groups were not significantly different from the rate in the UK human infants (Fig. 4). Simple contrasts with Chester as the referent revealed no significant group differences in face touching in Play (vs. PRI, *p* =.48; vs. Gombe, *p* =.56; vs. Aka, *p* =.23, Nso, *p* =.11).

In the context of Food beg/Food sharing, the Chester chimpanzee infants had a significantly higher rate of face touching than the UK human infants, *p* <.001, but all other groups were indistinguishable from the UK rates (Fig. 5). Simple contrasts with Chester as the referent revealed significant differences (all comparisons, *p* <.001).

In the context of Grooming/Being Groomed, the rate of face touching (with UK as referent) was significantly higher in two chimpanzee groups, Chester, *p* =.031, and PRI, *p* <.001, but all other groups were statistically indistinguishable from the UK human infants (Fig. 6). Simple contrasts with Chester as the referent revealed significant differences in face touching with Gombe, *p* =.04, Nso, *p* =.03, but no differences with PRI, *p* =.08; and Aka, *p* =.07.

**Fig. 7:**
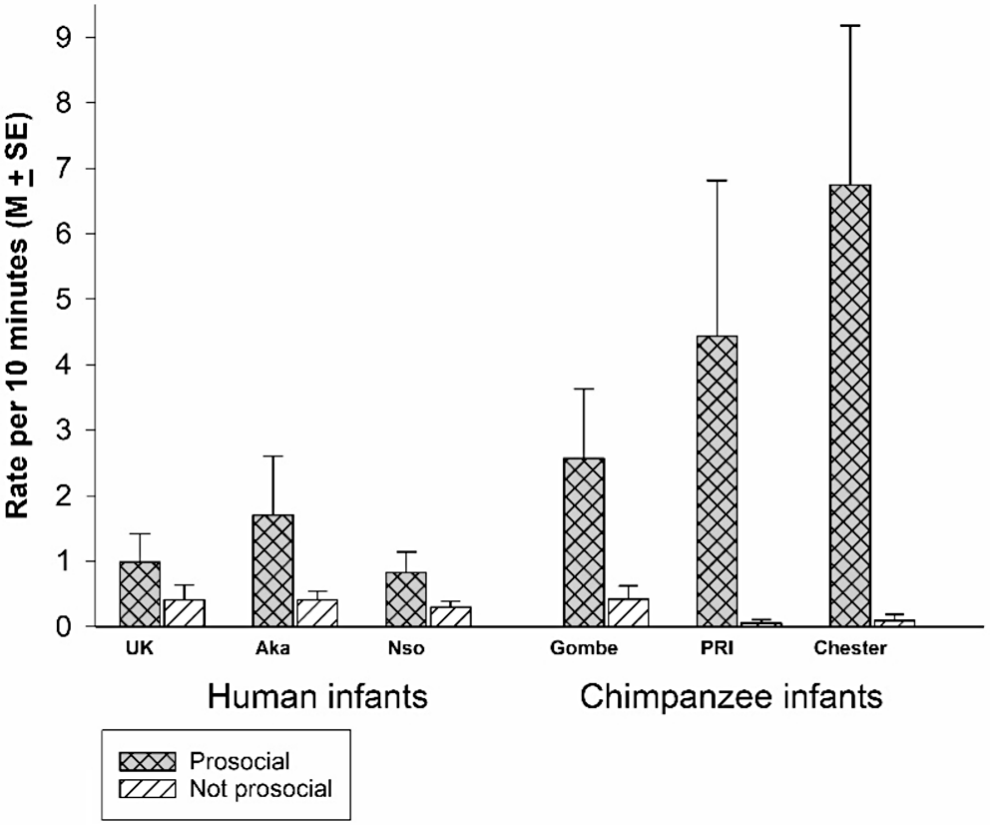
Rate of infants’ face touch on others in prosocial and non-prosocial contexts for each of the three groups of chimpanzees and the three groups of humans

Because there were relatively low rates of face touching in some of the individual contexts, we ran a new repeated measures ANOVA to compare the rate of face touching in all prosocial contexts versus the non-prosocial contexts. We found a significantly higher rate of face touch in prosocial compared to the non-prosocial contexts, *F*(1,45) = 30.313; *p* <.001; *eta*_*p*_^*2*^=0.402 (Fig. 7), an overall difference across groups, *F*(5,45) = 3.244, *p* =.014, *eta*_*p*_^*2*^ = 0.265, and a significant interaction, *F*(5,45) = 3.679, *p* =.007, *eta*_*p*_^*2*^ = 0.290.

To understand the group by context interaction, we ran ANOVAs separately for the two contexts. There was a significant difference among groups for prosocial contexts, *F*(5, 45) = 3.559; *p* =.009; *eta*_*p*_^*2*^=0.283. In simple contrasts (with the UK human group as the referent), we found a significant difference with Chester, *p* =.002, but no significant difference with any other groups (UK vs. PRI, *p* =.075; vs. Gombe, *p* =.22, vs. Aka, *p* =.59; vs. Nso, *p* =.98). In simple contrasts (with the Chester chimpanzee group as the referent), we found significant differences with all groups except PRI (*p* =.288): Chester vs. Gombe, *p* =.01, vs. Aka, *p* =.004; vs. Nso, *p* <.001; and vs. UK, *p* =.002). There was no significant difference among groups for the non-social contexts, *F*(5, 45) = 0.547; *p* =.740; *eta*_*p*_^*2*^=0.057.

### Face touching and triadic connectedness

**Fig. 8:**
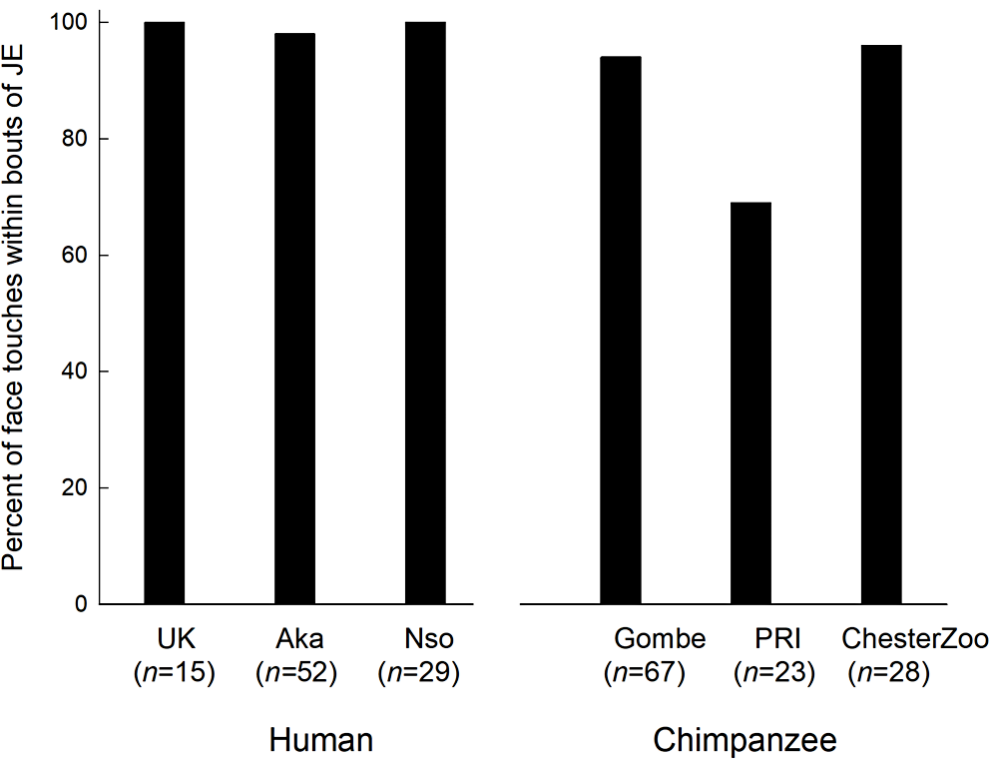
Percent of Face touches that occurred within bouts of Triadic Connectedness for each of the 3 groups of human infants and each of the 3 groups of chimpanzee infants (n is the total number of face touches per group)

The percentage of infant face touches that occurred within a bout of triadic connectedness is shown in Fig. 8. Chi-square tests, reported in Table 6 of the Supplemental Material 2, revealed that there were significantly more face touches occurring within bouts of triadic connectedness than would be expected in 5 groups, i.e., the UK human sample (*p* <.001), the Aka human sample (*p* <.001), the Nso human sample(*p* <.001), the Gombe chimpanzee sample (*p* <.001), and the Chester Zoo chimpanzee sample (*p* <.001). The chi-square for the PRI chimpanzees was a non-significant trend (*p* =.068).

## Discussion

One-year-old chimpanzee and human infants touched the face of others with their hands, during naturalistic interactions. These infants exhibited significantly higher rates of face touching than head touching (Fig. 2), and frequencies of face versus head touches differed by context and partner, suggesting that face touching has a function different from head touching or simple physical contact. Infant face touches were significantly associated with bouts of triadic connectedness (an inclusive form of joint attention) for five of the six infant groups. We interpret infant face touching to be a proximal marker of mutual engagement, an essential element of triadic connectedness.

Adult Females were the infants’ preferred partners for face touching (Fig. 3). Infant face-touching took place at significantly higher rates in prosocial contexts compared to non-prosocial contexts (Fig. 7). There was significant within group variation for the major contexts in which face touching occurred, i.e., play, grooming, and food beg/food sharing. In play, the Gombe chimpanzee infant engaged in significantly more face touching than the UK human infants. In Food beg/food share, the Chester chimpanzee infants face touched at the highest rate. In Groom/Being groomed, the Chester chimpanzee infants had higher rates of face touching than the Gombe chimpanzees, and the two non-UK groups of human infants. In these three contexts, the human groups did not significantly differ from each other, but their rates of face touching did not differ from at least one group of chimpanzee infants. Therefore, there was an overlap between species (chimpanzees and humans) for the contexts in which infants touch others’ faces. These analyses suggest that face touching serves a prosocial function that is preferentially directed to adult females (which includes mothers and other adult/adolescent females), but appears at different rates depending on the specific context.

In studies of mother-infant communication, mutual gaze and smiling are often considered to be ways that infants can mark their mutual engagement while they and a partner engage with objects (e.g., Akhtar and Gernsbacher 2008; Siposova and Carpenter 2019). Bakeman and Adamson (1984) used these markers to distinguish coordinated joint engagement from more passive forms of engagement. Bard (1990) suggested that the intentional communication (a type of coordinated joint engagement) of young orangutans in begging food from their mothers might be accompanied by touch markers, as gaze alternation is precluded when free-ranging infants ride on the neck/back of their mothers. Here we find that face touching overwhelming occurs during periods of triadic connectedness for both chimpanzee and human infants. Bard et al. (2021) broadened the definition of joint attention, using the term triadic connectedness, to be more inclusive of the range of cultural patterns of engagement with infants (e.g., proximal– emphasizing tactile communication, and distal– emphasizing visual communication: Keller et al. 2009; see Bard et al. 2021 for details of the joint attention coding and rationale for redefining joint attention as triadic connectedness). They suggested that there might be proximal markers (e.g., physical touches) of connectedness with partners during periods of triadic connectedness, as was found by chimpanzee mothers marking appropriate play faces in their young infants (Bard 1994). Here we found that the infant exhibited similar marking behavior in face touching, which appears to function in the same way as those maternal markers documented in western, middle-class human mothers with their infants (Adamson and Bakeman 1984). We are not suggesting that the only proximal marker of connectedness is face touching, but rather that one function of face touching, shared by human and chimpanzee infants, is as a marker of the infant’s engagement with their partner during bouts of triadic connectedness.

Infants’ engagement via face touching not only highlights the fact that infants can be active participants, but it may also allow them to regulate the interactions they receive (Botero 2016). Here we show that face touching has a specific function in pro-social contexts different from head-touching (noting that in humans, head touching is almost absent), similar to what was found in infant capuchin monkeys (Felicio et al. 2024). The results show that in chimpanzees and humans, the location of the touch (face or head) and the frequency and rate of face touches vary between contexts (especially in pro-social ones) and between interaction partners, with both human and chimpanzee infants, showing a general preference for face-touching. Infant face and head touches rarely occur in socially negative contexts. These results are similar to the study on capuchin monkeys (Felicio et al. 2024), except for the preference for partners. The chimpanzees and humans showed a preference for touching adult females, while capuchins had no preferences. The differences might be in how prevalent 1-year-old humans and 1-year-old chimpanzee engage with partners of different age/sex classes. Adult males were rarely touched by the UK and Nso humans and by the Gombe and PRI chimpanzees. In addition, the results indicate that there is a significant difference between groups, but not between species. A large variation within the species suggests that face-touching behavior varies according to the developmental and social environments of the infants. We also found that face-touching is associated with triadic connectedness events, so this behavior could retain attention and encourage the maintenance of social engagement.

Therefore, face-touching performed by infant primates may confer an advantage for their social development and engagement, similar to how grooming (Jablonski 2020) and lip-smacking (Albuquerque et al. 2023) reinforce social bonds and appear in various primate species, but face touching may be more focused on the realm of social cognition. Social touch, in particular grooming, is fundamental for the evolution of social cohesion in primates and, summed with the salient social information of the context, permits social allostasis, which maintains long-term bonds (Jablonski 2020). Bard (2009) suggests that touch occurs throughout primates within social cognition contexts, as proximal parenting is more prevalent than the distal parenting styles that emphasize gaze. This active touch by infants aligns with the importance of touch for primate social life and infant development. Our findings make evident the need to pay attention to tactile interactions that might be less conspicuous, especially those initiated by the infant since we can mistakenly assume that they have a more passive role.

In conclusion, face-touching behavior by 1-year-old chimpanzees and humans is associated with positive prosocial contexts. In these samples, there was a preference for face-touching over head-touching, which occurred in different contexts (perhaps suggesting different social functions) and a preference for touching the faces of adult and adolescent females over others. By confirming the presence of this behavior in both species and in all groups, we can say that face-touching by infants is part of their social repertoire. Moreover, we suggest that face touching may have a particular cognitive function in great apes because it occurs overwhelmingly in the context of triadic connectedness, an early appearing type of social cognition.

We suggest that the social functions of face touching could be adaptive, given that infants’ face-touching of other individuals had a significant impact in the three primate species studied to date (chimpanzees and humans in the current study and capuchins in Felicio et al. 2024). Its broad association with prosocial contexts, even though the groups differ from each other within some contexts, supports the argument that face touching has positive functions for initiating and/or maintaining coordinated engagement. These findings show that primate infants can take an active role in initiating or maintaining their social engagements through face touching and great ape infants can use this behavior as a marker of mutual engagement during bouts of triadic connectedness (an inclusive form of joint attention).

## Electronic supplementary material

Below is the link to the electronic supplementary material.

Supplementary Material 1

Supplementary Material 2

## Data Availability

Data is provided within the manuscript or supplementary information files.
