# Supplementary material for: Great ape infants’ face touching and its role in social engagement
Source: Anim Cogn. 2025 Feb 5;28(1):10. doi: 10.1007/s10071-025-01931-8 (PMC11799006; doi:10.1007/s10071-025-01931-8)
Supplement: Supplementary file 2 — Supplementary Material 2 [file 10071_2025_1931_MOESM2_ESM.docx]

**Title: Great Ape infants’ face touching and its role in social engagement**

**Journal: Animal Cognition**

**Authors:** Beatriz Felicio, Kim A. Bard

**Corresponding author:** beatriz.franco.santos@usp.br

Supplemental Table 1

Detailed locations of infant face and head touches to social partners

Face Head

Detailed location

Crown 0 8

Forehead/head 0 1

Ear 1 28

Side 3 4

More than 1location 34 4

Forehead 6 1

Forehead/eyes 2 0

Cheek 45 0

Cheek/chin 1 0

Chin 15 0

Eye 1 0

Eyes/nose 2 0

Jaw 9 0

Mouth 75 1

Mouth/cheek 2 0

Mouth/Chin 4 0

Nose 16 0

Nose/mouth 3 0

Detail Not visible 3 0

Total 222 47 (269)

Supplemental Table 2

Frequency counts (expected frequencies) of touch location in each of 7 contexts (Bold indicates the 4 cells with expected frequencies less than 5, when only 2 were permissible).

Touch location

Context Face Head

Play 97 (99.1) 24 (21.9)

Food beg/Food share 24 (27.8) 0 **( 4.3)**

Groom/Being groomed 13 (14.7) 5 **( 3.3)**

Prosocial Rare 12 ( 9.8) 0 **( 2.2)**

Other Social 25 (27.8) 9 ( 6.2)

Negative-social 10 ( 9.0) 1 **( 2.0)**

Non-social 27 (27.8) 7 ( 6.2)

Supplemental Table 3

Frequency counts (expected frequencies) of touch location in each of 5 contexts.

Touch location

Contexts (reduced) Face Head

Play 97 (99.1) 24 (21.9)

Food beg/Food share 24 (19.7) 0 ( 4.3)

Other social 50 (52.4) 14 (11.6)

Negative-social 10 ( 9.0) 1 ( 2.0)

Non-social 27 (27.8) 7 ( 6.2)

Supplemental Table 4

Details about the scoring of touch partners (first column) and the 4 categories used in analyses (columns 2-5). Entries are frequencies, x indicate absence of opportunity, 0 indicates absence of occurrence.

Group Partner Code

(as scored) AdultFem AdMale Juvenile/child Infant Total

Aka (Human)

Adult female 18 0 0 0 18

Adult male 0 24 0 0 24

Child 0 0 4 0 4

Infant 0 0 0 3 3

Total 18 24 4 3 49

Chester Zoo (chimpanzee)

Mother 30 0 x 0 30

Adult female 1 0 x 0 1

Adult male 0 3 x 0 3

Infant 0 0 x 3 3

Total 31 3 x 3 37

Gombe (chimpanzee)

Mother 63 0 0 0 63

Sibling’s name 0 0 14 0 14

Partner’sname 0 0 2 0 2

Infant 0 0 0 19 19

Total 63 0 16 19 98

Nso (human)

Adult female 18 0 0 0 18

Adolescent 3 0 0 0 3

Juvenile 0 0 6 0 6

Child 0 0 6 0 6

Infant 0 0 0 2 2

Total 21 0 12 2 35

PRI (chimpanzee)

Adult female 20 0 x 0 10

Infant 0 0 x 13

Total 20 0 x 13 33

UK (human)

Mother 13 0 0 x 13

Father 0 3 0 x 3

Older sister 0 0 1 1

Total 13 3 1 x 17

Total 166 30 33 40 269

Supplemental Table 5

Frequency counts (and expected frequencies) of touch location for each of 4 partner types

Touch location

Partners (reduced) Face Head

Adult Female 146 (142.5) 28 (31.5)

Adult Male 30 ( 24.6) 0 ( 5.4)

Juvenile 10 ( 8.2) 0 ( 1.8)

Infant 22 ( 32.8) 18 ( 7.2)

Supplemental Table 6

Chi-square matrices and statistical results for whether face touching occurred with triadic engagement more than expected in each of the 3 human groups and the 3 chimpanzee groups.

HUMAN GROUPS

**Aka** Triadic Connectedness *Chi-square*(1)=22.785, *p*<.001

Yes No

Face Yes 51 1

Touch No 1356 678

**Nso** Triadic Connectedness *Chi-square*(1)=12.08, *p*<.001

Yes No

Face Yes 29 0

Touch No 1415 593

**UK** Triadic Connectedness *Chi-square*(1)=11.01, *p*<.001

Yes No

Face Yes 15 0

Touch No 953 704

CHIMPANZEE GROUPS

**Gombe** Triadic Connectedness *Chi-square*(1)=24.675, *p*<.001

Yes No

Face Yes 63 4

Touch No 735 408

**PRI** Triadic Connectedness *Chi-square*(1)=3.31, *p=.068*

Yes No

Face Yes 16 7

Touch No 391 379

**Chester** Triadic Connectedness *Chi-square*(1)=12.14, *p*<.001

Yes No

Face Yes 27 1

Touch No 343 176
